# Spectroelectrochemical Behavior of Polycrystalline Gold Electrode Modified by Reverse Micelles

**DOI:** 10.3390/molecules26020471

**Published:** 2021-01-18

**Authors:** Miriam C. Rodríguez González, Maximina Luis-Sunga, Ricardo M. Souto, Alberto Hernández Creus, Elena Pastor, Gonzalo García

**Affiliations:** Instituto de Materiales y Nanotecnología, Departamento de Química, Universidad de La Laguna, PO Box 456, 38200 La Laguna, Spain; mrodrgon@ull.edu.es (M.C.R.G.); mluissun@ull.edu.es (M.L.-S.); rsouto@ull.es (R.M.S.); ahcreus@ull.edu.es (A.H.C.); epastor@ull.edu.es (E.P.)

**Keywords:** gold, adsorption, surfactant, micelles, AFM, EIS, electron tunneling

## Abstract

The increasing demand for raising the reliability of electronic contacts has led to the development of methods that protect metal surfaces against atmospheric corrosion agents. This severe problem implies an important economic cost annually but small amounts of corrosion inhibitors can control, decrease or avoid reactions between a metal and its environment. In this regard, surfactant inhibitors have displayed many advantages such as low price, easy fabrication, low toxicity and high inhibition efficiency. For this reason, in this article, the spectroelectrochemical behavior of polycrystalline gold electrode modified by reverse micelles (water/polyethyleneglycol-dodecylether (BRIJ 30)/*n*-heptane) is investigated by atomic force microscopy (AFM), potentiodynamic methods and electrochemical impedance spectroscopy (EIS). Main results indicate a strong adsorption of a monolayer of micelles on the gold substrate in which electron tunneling conduction is still possible. Therefore, this method of increasing the corrosion resistance of gold contacts is usable only in conditions of long-term storage but not in the operation of devices with such contacts. In this regard, the micelle coating must be removed from the surface of the gold contacts before use. Finally, the aim of the present work is to understand the reactions occurring at the surfactant/metal interface, which may help to improve the fabrication of novel electrodes.

## 1. Introduction

Electrical contact is defined as the interface between the current-carrying members of an electrical/electronic device. Its goal is to allow a continuous passage of electrical current through the interface, which can only be achieved if it is established a good metal-to-metal contact [1]. Electronic connectors and many other contacts must function in chemically aggressive environments [2]. Therefore, the need to ensure and maintain a long lifetime of the electronic connections under severe operation conditions has been emphasized. The preferred contact materials for electronic connectors, which are used primarily as electrodeposits and coatings, are precious metals such as gold, palladium and their alloys [1].

In this regard, gold materials have received increasing interest in many fields of research due to their excellent physical and chemical properties [3]. Gold is a noble metal and plays an important role in nanoscience and nanotechnology because of its high stability at the nanoscale [4]. Corrosion of gold can only take place in the presence of strong oxidants agents and complexing ions. Gold may suffer corrosion and even dissolve in solutions containing complexing agents [3]. This phenomenon is relevant in the electronics industry due to its use in thin-film applications and integrated-circuit technology [5]. Also, the addition of copper, palladium, silver or platinum obtaining binary and ternary alloys may improve hardness without losing tarnish resistance but its use is restricted to low current applications [1]. Among the plating materials, gold has the highest standard electrode potential (E^0^ = 1.38 V). When thin, precious metal finishes, most commonly gold, are used the corrosion is originated by intrinsic porosity, wear tracks and occasionally defects caused by forming and handling during the manufacturing process [2]. A perfect gold coating does not present corrosion problems; however, due to the high cost of this material, the gold plating is usually so thin that the final coating is not free of pores. These pores cause corrosion of electrically conductive surfaces with protective coatings [6].

One way of protecting against the corrosion of conductive surfaces consists of passivation. In this process, the metal surface is changed creating resistance to environmental agents. Metal surfaces can be passivated by their own oxide layer or by a non-reactive layer such as self-organizing nanoparticles. In addition to resisting corrosion, the conductive surfaces must meet two important requirements: (i) that the passivation layer must not significantly increase or destabilize the electrical resistance and (ii) the passivation layer must withstand the operating temperature (which will be the sum of the resistive heating and ambient temperature) [6]. The use of corrosion inhibitors is one of the most effective methods for protection against corrosion in an acid medium. A typical inhibitor should: (i) remove water from the metal surface, (ii) interact with the cathodic or anodic reaction sites to inhibit the corresponding redox reaction and (iii) impede the transport of water and corrosive species to the metal surface [6,7].

Organic compounds are widely used in industry as inhibitors for acidic media. This type of compounds may contain multiple bonds with nitrogen, sulfur and oxygen atoms through which they can be adsorbed on the metal surface [7]. It is expected to found efficient inhibitors that can function in a wide range of parameters, considering that the effectiveness of the inhibition depends on the conditions of the system (temperature, pH, the composition of the material and duration) and on the structure of the inhibitor compound [8]. In this regard, surfactant inhibitors have great advantages such as high inhibition efficiency, low cost, easy production and low toxicity. Besides, the study of surfactant substances adsorbed on metallic surfaces is important for electrochemical studies such as the inhibition of corrosion and adhesion phenomena. It is necessary that the surfactant functional group is adsorbed onto the metal surface to achieve corrosion inhibition. The degree of adsorption depends on the surface structure, the nature of the metal, the chemical nature of the inhibitor, the adsorption mode and the type of media. Metal corrosion will be inhibited by adsorbing organic molecules or ions on the surface forming a protective layer that will reduce or prevent metallic oxidation [8,9].

Hence, adsorption is critical and its capacity is generally related to the ability to aggregate to form micelles [8]. One of the better established ways to obtain micelles is the microemulsion technique. In this process, small amounts of water are added to a surfactant/oil solution, obtaining a mixture known as a water-in-oil microemulsion [10]. The molecules of surfactants are amphiphilic, with a hydrophilic head and a hydrophobic tail and therefore they are easily adsorbed at interfaces. At low concentrations, they mainly exist as individual molecules but as the concentration increases and the critical micellar concentration (CMC) is reached, the surfactant molecules create the so-called micelles. In aqueous solution, the hydrophobic tails are found within the micelle and only the hydrophilic part is exposed to the aqueous phase [11]. 

The promising potential application of micelles as corrosion inhibitors can lead to a better understanding of the relationship between the adsorption of the surfactant molecules and the metal surface helping to improve the corrosion inhibition, which would be a step forward to the improvement in the protection of electrical contacts.

## 2. Results and Discussion

### 2.1. Atomic Force Microscopy (AFM) Characterization

**Figure 1:**
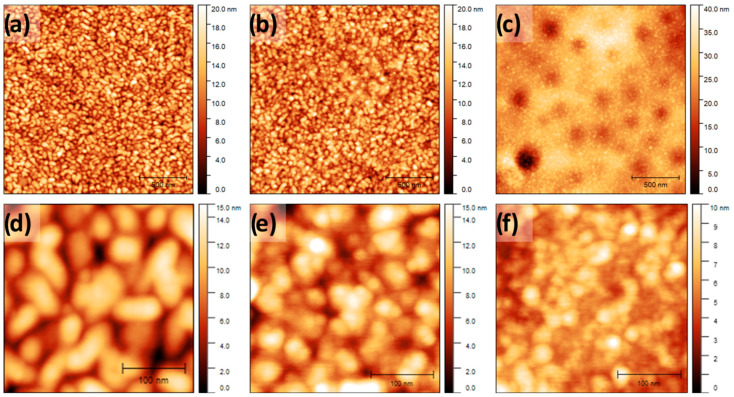
Atomic force microscopy (AFM) topographic images of 2 µm × 2 µm (**a**–**c**) and 0.6 µm × 0.6 µm (**d**–**f**) for bare polycrystalline gold substrate (**a**,**d**), monolayer of micelles (**b**,**e**) and multilayer of micelles (**c**,**f**).

Figure 1 depicts the AFM analysis for the samples prepared. Figure 1a,d show the bare substrate prior to any modification. A surface formed by gold grains of around 50 nm can be found. Multilayer of micelles with diameter close to 20 nm was formed on the gold surface after deposition of 20 µL of micelles-containing solution (Figure 1c,f). After that, the sample was copiously rinsed with acetone and ultra-pure water and a monolayer of circular features of ca. 20 nm in diameter adsorbed on the gold grains previously observed in Figure 1a,d are discerned (Figure 1b,e). The latter indicates strong adsorption strength of a monolayer of micelles onto the gold substrate and that the interaction energy between the gold surface and the micelles is higher than that between water, heptane and acetone molecules and the gold substrate.

The previous is supported by DLS measurements that indicate a micelle average size of ca. 14 nm in solution (Figure S1), which increases to 20 nm in diameter with 1.4 nm in thickness (Figure S2) after micelles deposition onto gold, that is, micelles flatten in contact with gold due to the strong interaction between them. In this sense, it is remarkable the stability of the adsorbed monolayer of micelles on the gold surface that remains unchanged after long exposure (~7 days) into acidic (1 M H_2_SO_4_), alkaline (1 M NaOH) and organic (heptane) media. 

The observations in Figure 1 can be easily explained by the critical micelle concentration (CMC), which is a key factor in determining the effectiveness of a corrosion inhibitor [8]. Above the CMC the gold surface is covered with a monolayer of micelles and the additional molecules combine to form micelles or multiple layers (Figure 1c,f) [8]. The additional organic molecules are easily removed by washing with acetone and water, leaving a strong adsorbed monolayer film of micelles on the gold surface (Figure 1b,e).

Surface roughness (RMS) data for small-scale images (Figure 1d–f) confirm the gold surface coverage by micelles. Indeed, RMS values of 2.819, 2.556 and 1.739 nm were found for the bare substrate, a monolayer and multilayer of micelles, respectively. The last indicates the formation of smooth mono/multilayer of micelles onto the gold substrate with a subsequent decrease in roughness of the surface upon adsorption.

Since multilayer of micelles are not strongly adsorbed on the gold substrate, electrochemical and EIS experiments of bare gold and gold-covered by a monolayer of micelles are described, compared and analyzed in the following subsections.

### 2.2. Electrochemical Characterization of Micelle Monolayer Formed on Gold

A comparative analysis of the electrochemical behavior of the micelle-coated gold surface and the pristine electrode was performed by considering simple one-electron transfer reactions involving a change in the oxidation state of iron, namely hexacyanoferrate(III), [Fe(CN)_6_]^3−^, and ferrocenemethanol, FcMeOH, in 0.1 M KCl solution. The iron complexes were chosen as to consider both an outer-sphere redox system, FcMeOH^1+/0^, and an inner-sphere redox pair, [Fe(CN)_6_]^3−/4−^. Measurements were initiated after 60 min immersion in the solution to attain their corresponding open circuit potentials: +0.165 and +0.090 V vs. SCE, respectively. No significant potential differences (namely, less than 3 mV) were observed within the OCP values displayed by the pristine and the modified gold surfaces for each given redox mediator system.

**Figure 2:**
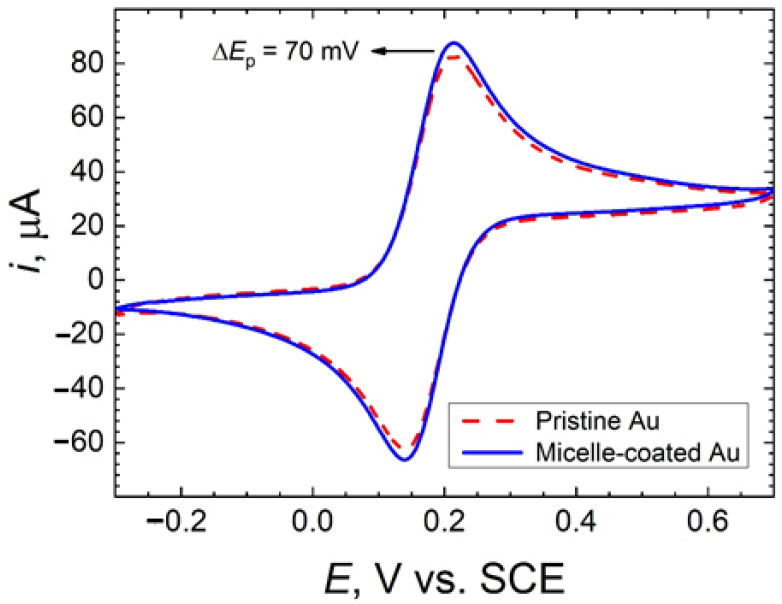
Cyclic voltammogram for pristine and micelle-coated gold electrodes in 0.1 M KCl + 1 mM ferrocenemethanol. Scan rate: 20 mV s^−1^; Area: 0.5 cm^2^.

**Figure 3:**
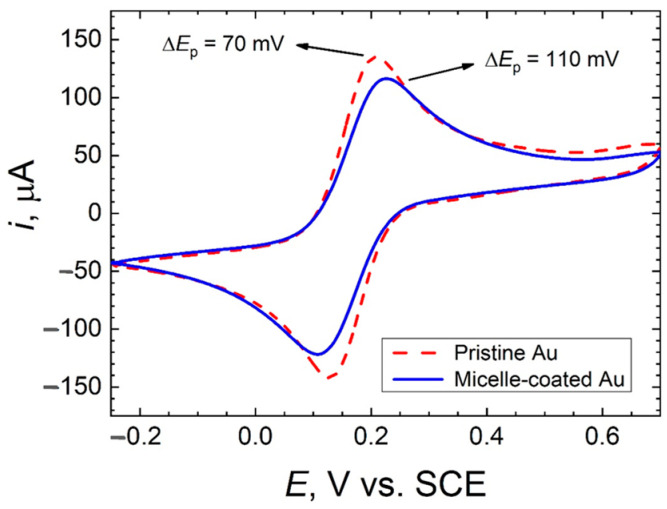
Cyclic voltammogram for pristine and micelle modified gold in 0.1 M KCl + 1 mM K_3_Fe(CN)_6_. Scan rate: 20 mV s^−1^; Area: 0.5 cm^2^.

Figure 2 and Figure 3 display the cyclic voltammograms recorded for pristine gold and micelle-modified gold electrodes immersed in 0.1 M KCl solution containing 1 mM concentration of either ferrocenemethanol or hexacyanoferrate(III). A well-defined (quasi-)reversible system is observed in all cases, although the attenuation effect due to surface modification by micelles greatly depended on the nature of the redox mediator species.

As expected, voltammetric profiles of both electrodes in 0.1 M KCl solution containing ferrocenemethanol are approximately equal (Figure 2). On the other hand, the presence of a monolayer of micelles on the gold electrode in 0.1 M KCl solution containing hexacyanoferrate(III) increases the irreversibility of the systems (i.e., faradaic peaks become more separated) and slightly decreases the active surface area (Figure 3). Indeed, the active surface area decreases about 10% after modification of the gold electrode by a monolayer of micelles. Interestingly, the latter is in agreement with AFM analysis, in which a surface roughness (RMS) reduction of 10% was discerned (see Section 2.1).

### 2.3. Electrochemical Impedance Spectra of Micelle Monolayer Formed on Gold

**Figure 4:**
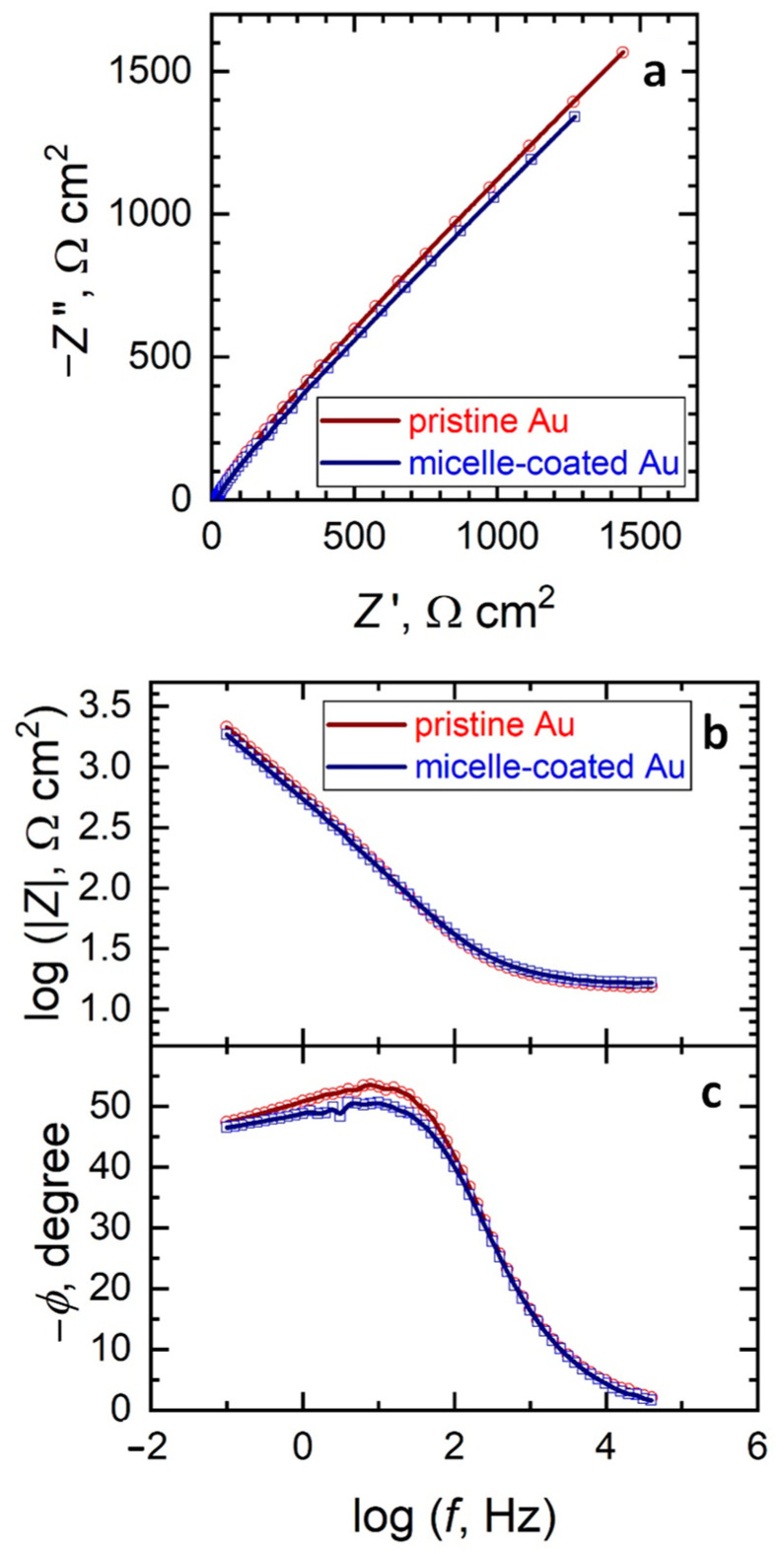
(**a**) Nyquist and (**b**,**c**) Bode diagrams of the impedance spectra of pristine and micelle-modified gold in 0.1 M KCl + 1 mM ferrocenemethanol. The solid lines and the discrete points correspond to the fitted and the measured data, respectively.

**Figure 5:**
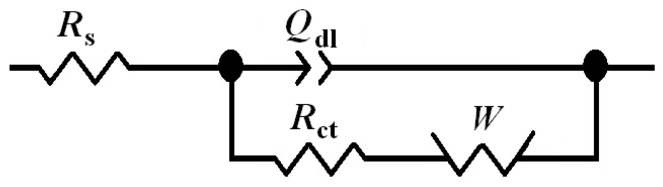
Equivalent electrical circuit employed to fit the electrochemical impedance spectroscopy (EIS) spectra.

The assessment of the different electrochemical reactivity of the micelle-coated gold surface for each redox mediator is more readily observable using electrochemical impedance spectroscopy (EIS). Figure 4 depicts the Nyquist and Bode impedance plots of pristine and micelle-coated gold for the redox conversion of ferrocenemethanol. As can be seen from the Nyquist plots shown in Figure 4a, similar impedance spectra were obtained in both cases. They display one time constant and they can be satisfactorily fitted using the Randles-Ershler equivalent circuit shown in Figure 5, where *R*s relates to the resistance of the solutions, *R*_ct_ corresponds to the electron transfer reaction, *Q*_dl_ to the capacitive response of the double layer and *W* accounts for the diffusion of the soluble redox species. One constant phase element (CPE) was employed instead of a capacitor, owing to reported dispersion effects produced by microscopic roughness of the metal surface [12]. Therefore, the redox process is kinetically controlled in the high frequency range, whereas the Warburg impedance dominates at lower frequencies. The system shows no apparent heterogeneities and the redox reaction can be described to occur homogeneously on the surfaces of both pristine and micelle-coated gold electrodes of apparently equal areas. This result agrees well with the cyclic voltammetry study (see Section 2.2) and with the assumption that an outer-sphere system does not involve specific surface adsorption for the electron transfer process to occur [13]. Therefore, similar behaviors are recorded for the pristine and the micelle-coated gold electrodes for the redox conversion of ferrocenemethanol.

**Figure 6:**
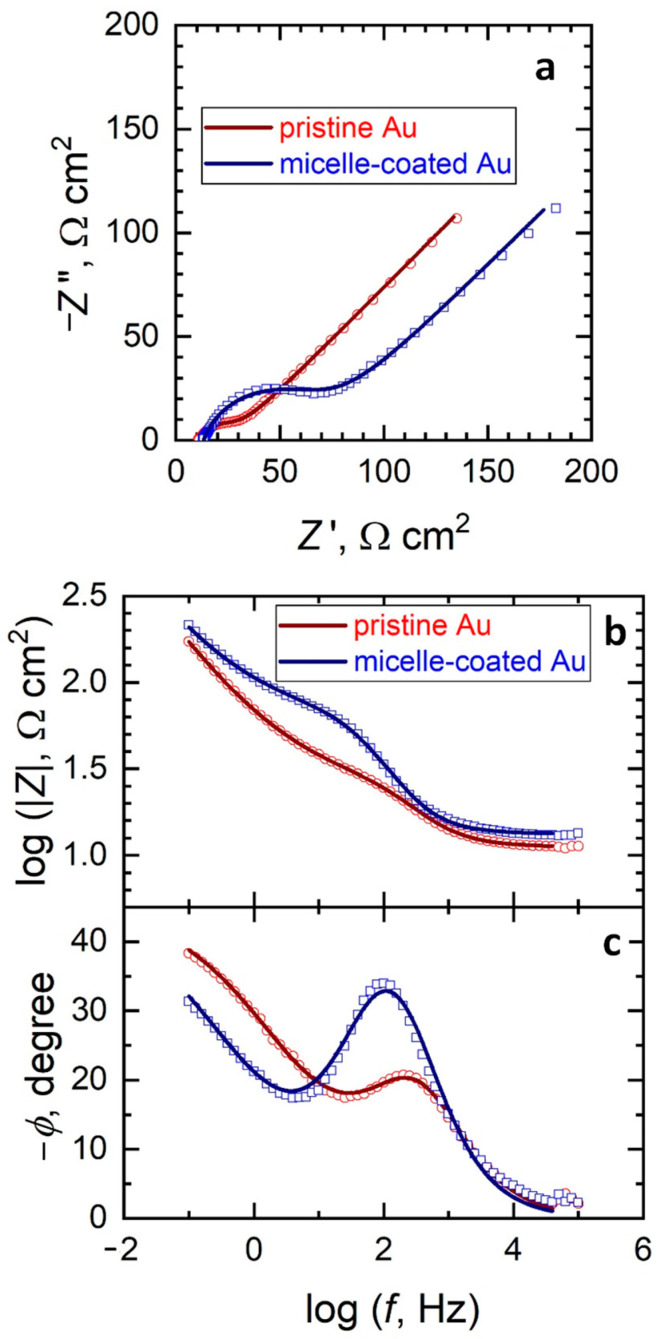
(**a**) Nyquist and (**b**,**c**) Bode diagrams of the impedance spectra of pristine and micelle-modified gold in 0.1 M KCl + 1 mM K_3_Fe(CN)_6_. The solid lines and the discrete points correspond to the fitted and the measured data, respectively.

When hexacyanoferrate was used as redox mediator the heterogeneous electron transfer occurred significantly faster than in the case of ferrocenemethanol as shown by the impedance spectra in Figure 6. In addition, the Nyquist and Bode plots for this redox mediator were observed to clearly separate between them for the pristine and the micelle-coated gold electrodes, due to marked decrease in the reversibility of the heterogeneous electron transfer (HET) due to the occurrence of the surface film formed by the micelle layer on the surface of gold (Figure 6). Yet, the spectra could be satisfactorily fitted by the Randles-Ershler equivalent circuit for both the pristine and the micelle-coated gold electrodes as described by the equivalent circuit shown in Figure 5 and the obtained impedance parameters are also included in Table 1. This feature suggests that the main effect of the micelle layer on the HET process is to promote some degree of irreversibility in the otherwise fast electron transfer reaction associated to the reduction of hexacyanoferrate(III). 

Table 1 lists the impedance parameters used in the modelling of the impedance spectra given in Figure 4 and Figure 6. It is interesting to notice that a 3-fold increase of the charge transfer resistance *R*_ct_ occurred when the HET for the reduction of hexacyanoferrate(III) occurred on the micelle-coated Au surface compared to the pristine metal condition, whereas all the remaining parameters showed only minor variations between the two conditions, even for the *R*_ct_ values determined for the ferrocenemethanol redox system. In summary, electrochemical impedance data agree well with the observations previously derived from the electrochemical characterization using cyclic voltammetry and the surface analytical findings revealed by AFM imaging.

## 3. Materials and Methods

### 3.1. Preparation of the Material

Reverse micelles were obtained by the microemulsion method previously described in [10,14,15]. Water/oil microemulsion comprised of water/polyethyleneglycol-dodecylether (BRIJ 30)/*n*-heptane was used to obtain the reverse micelles. The size of the micelles is determined by the molar ratio (R) of water to surfactant that is six in the current work. The size of the micelles is corroborated by dynamic light scattering (DLS) measurements on a Malvern Zetasizer Nano S (Malvern Instruments Ltd., Malvern, UK) with a detection angle of 173°. All measurements in this study were taken at a temperature of 25 °C and at least 3 repeat measurements were taken to check for result repeatability. The intensity size distributions were obtained from analysis of the correlation functions using the algorithm in the instrument software. Figure S1 reveals a monodisperse sample with micelle average size close to 14 nm.

For the preparation of the modified gold surface, polycrystalline Au plates (Geometric area ~0.5 cm^2^; Arrandee, Werther, Germany) were cleaned by the ultrasound method (first with acetone and then with ultra-pure water) and used as the substrate. Then, 20 µL of the micellar solution is drop-casted on the clean polycrystalline gold substrate. In order to obtain a monolayer of micelles containing water onto the gold surface, the sample was thoroughly rinsed with acetone and ultra-pure water to remove the excess of the organic material.

### 3.2. Atomic Force Microscopy (AFM) Analysis

Topographic atomic force microscopy (AFM) images were acquired in Peak-Force and Tapping modes using a multimode microscope with a Nanoscope V control unit from Bruker. Scan rates of 0.5–1.2 Hz were used. Measurements were done with Scan-Asyst HR (50–100 kHz and 1–5 N m^−1^) tips (Bruker). AFM images were analyzed with Gwyddion software [16].

### 3.3. Electrochemical Characterization of the Material

The electrochemical tests were conducted using an AUTOLAB PGSTAT302N electrochemical workstation (Metrohm Autolab bv, Utrecht, The Netherlands) in the standard three electrode configuration, wherein a saturated calomel electrode (SCE) and a gold grid were employed as the reference and the counter electrodes, respectively. The pristine gold electrode or micelle-coated gold electrode served as the working electrode (surface area, 0.5 cm^2^). Before each experiment, the working electrode was immersed in a 0.1 M KCl test solution containing 1 mM concentration of a reversible redox mediator for 60 min to attain a steady state open circuit potential (OCP). Potassium hexacyanoferrate (III) (99%, Sigma-Aldrich; St. Louis, MO, USA) and ferrocenemethanol (97%, Sigma-Aldrich; St. Louis, MO, USA) were employed as redox mediators to characterize the reactivity of the working electrode towards the electron transfer reaction. Solutions were prepared using pro analysis reactants and ultrapure deionized water (resistivity = 18.2 MΩ cm, Milli-Q; Millipore, Burlington, MA, USA). Tests were performed in the de-aerated test solutions at the laboratory temperature (20 ± 2 °C).

Electrochemical impedance spectra (EIS) were obtained using a perturbation amplitude of ±10 mV (vs. OCP) and a frequency interval from 100 kHz to 100 mHz. EIS data were analyzed using Yeum’s ZsimpWin 2.00 software and fitted to electrical equivalent circuits. The impedance data were represented in terms of both Nyquist (imaginary component of the impedance as a function of the real component) and Bode (logarithm of the impedance modulus, |*Z*| and phase angle (*φ*) as a function of the logarithm of the frequency (*f*) plots. Cyclic voltammograms (CV) were recorded in the range from −0.25 to +0.70 V vs. SCE with a scan rate of 20 mV s^−1^.

## 4. Conclusions

In this study, the influence of reverse micelles (water/polyethyleneglycol-dodecylether/*n*-heptane; R = 6) on the corrosion behavior of polycrystalline gold in 0.1 M KCl solution was investigated using atomic force microscopy (AFM), cyclic voltammetry and electrochemical impedance spectroscopy (EIS) techniques.

AFM images showed a smooth monolayer of micelles strongly adsorbed on the gold surface, which decreases the surface roughness (RMS) of the bare electrode about 10%. In this sense, electrochemical experiments performed with the inner-sphere redox pair species revealed that the active surface area also decreases around 10% after gold modification by a monolayer of micelles, although a decrease in the reversibility of the redox system was discerned. The latter was confirmed by EIS experiments in which the charge transfer resistance rises after gold modification by a monolayer of micelles. Furthermore, EIS results indicated the Randles-Ershler equivalent circuit as the operative in the presence and the absence of micelles on the gold electrode, which indicates a thin film of micelles covering the gold surface.

All in all, the outcomes suggest a compact thin film (1.4 nm thickness) of micelles strongly adsorbed on the gold electrode that only allows electron tunneling through the organic barrier. This consequence would be more beneficial for metal protection in conditions of long-term storage, since molecular/metal species are impeded to pass through the physical barrier but electron tunneling conduction is still possible.

## Figures and Tables

**Table 1:** Electrochemical parameters obtained from EIS data measured for pristine and micelle-modified gold for the heterogeneous electron transfer (HET) of ferrocenemethanol and hexacyanoferrate(III). The meaning of the electrochemical parameters is given by the equivalent circuit shown in Figure 5.

Impedance Parameter	Redox Mediator			
	Ferrocenemethanol		Hexacyanoferrate(III)	
	Pristine Au	Micelle-Coated Au	Pristine Au	Micelle-Coated Au
*R*_s_, Ω cm^2^	15.36	16.30	11.12	13.22
*Q*_dl_, mS cm^−2^ s*^n^*	0.3661	0.4016	0.2805	0.2009
*n*_dl_	0.6953	0.6769	0.7507	0.7939
*R*_ct_, Ω cm^2^	1372	1005	19.01	58.62
*W*, Ω cm^2^ s^−1^	9.612 × 10^−5^	1.087 × 10^−4^	2.048 × 10^−4^	2.016 × 10^−4^

## Data Availability

The data presented in this study are available in the current work.

## Supplementary Materials

The following are available online, Figure S1: Size distribution by intensity of reverse micelles in *n*-heptane solution. Average size = 13.96 nm. Figure S2. 600 × 200 nm^2^ AFM image showing the topography of a micelle layer on an Au(111) surface bearing a 400 × 100 nm^2^ scratch made in a smooth Au(111) terrace (a). Depth profile histogram exhibiting the depth value distributions related (calculated from the region marked by the white-dashed rectangle) to bare gold, blue line and the micelles, red (b). From the height difference between the later, the thickness of the film, that is 1.4 nm, can be obtained.

## Sample Availability

Samples of the micelles are available from the authors.
