# Supplementary material for: Spectroelectrochemical Behavior of Polycrystalline Gold Electrode Modified by Reverse Micelles
Source: Molecules. 2021 Jan 18;26(2):471. doi: 10.3390/molecules26020471 (PMC7830569; doi:10.3390/molecules26020471)
Supplement: Supplementary file 1 [file molecules-26-00471-s001.pdf]

Supporting Information

Spectroelectrochemical behavior of polycrystalline gold electrode modified by reverse micelles

Miriam C. Rodríguez González, Maximina Luis Sunga, Ricardo M. Souto, Alberto Hernández Creus, Elena Pastor and Gonzalo García*

Instituto de Materiales y Nanotecnología, Departamento de Química, Universidad de La Laguna, PO Box 456, 38200, La Laguna, Santa Cruz de Tenerife, Spain *****

*Correspondence: ggarcia@ull.edu.es

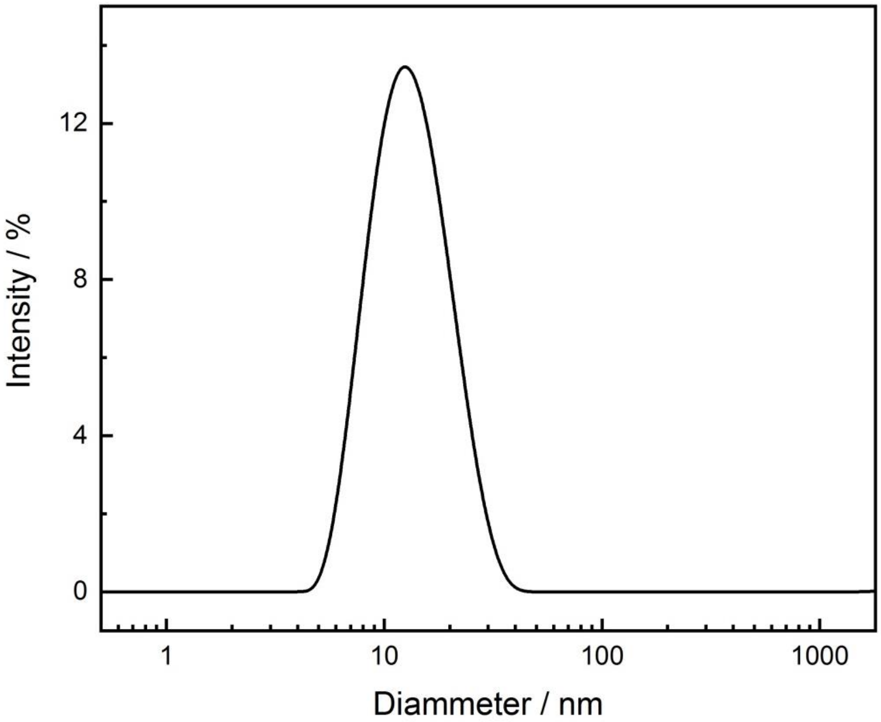

Figure S1. Size distribution by intensity of reverse micelles in n-heptane solution. Average size = 13.96 nm.

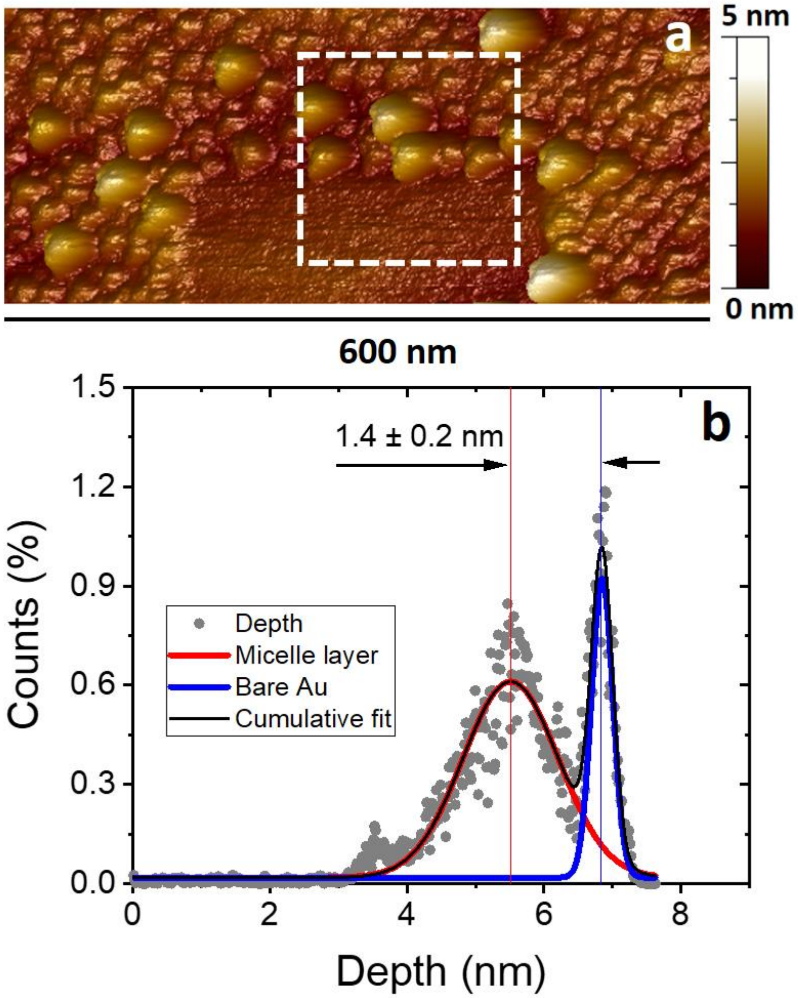

Figure S2. 600 x 200 nm^2 AFM image showing the topography of a micelle layer on an Au(111) surface bearing a 400 x 100 nm^2 scratch made in a smooth Au(111) terrace (a). Depth profile histogram exhibiting the depth value distributions related (calculated from the region marked by the white-dashed rectangle) to bare gold, blue line, and the micelles, red (b). From the height difference between the later, the thickness of the film, i.e. 1.4 nm, can be obtained.
